# Illness and Injury Burdens Among Reserve Component Members of the U.S. Armed Forces, 2024

**Published:** 2025-09-20

**FIGURE 1.**
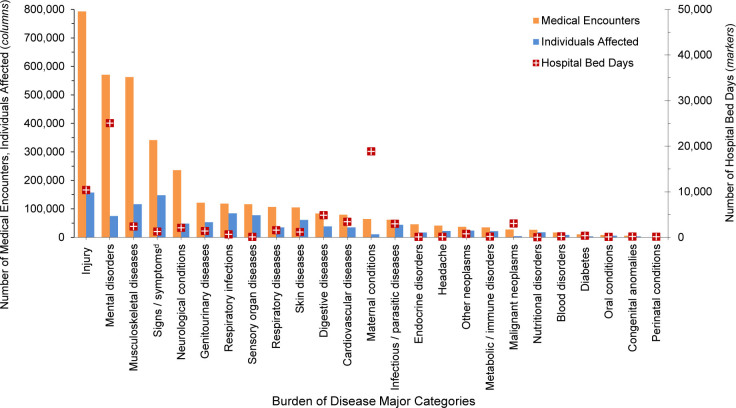
Numbers of Medical Encounters ^a^ , Individuals Affected and Hospital Bed Days by Burden of Disease Major Category ^b^ , Reserve Componentd, U.S. Armed Forces, 2024

**FIGURE 2.**
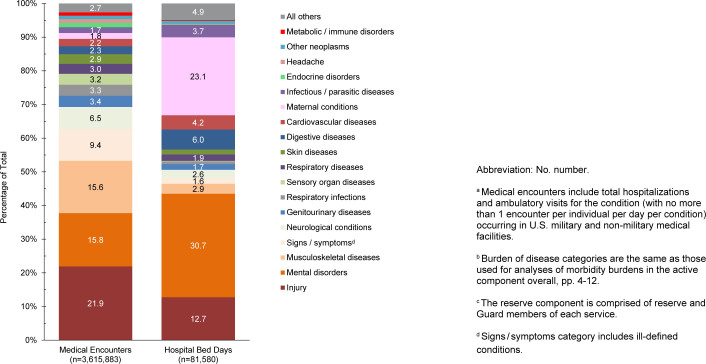
Percentages of Medical Encounters ^a^ and Hospital Bed Days by Burden of Disease Category ^b^ , Reserve Component ^c^ , U.S. Armed Forces, 2024

